# Generation of the first BAC-based physical map of the common carp genome

**DOI:** 10.1186/1471-2164-12-537

**Published:** 2011-11-02

**Authors:** Peng Xu, Jian Wang, Jintu Wang, Runzi Cui, Yan Li, Zixia Zhao, Peifeng Ji, Yan Zhang, Jiongtang Li, Xiaowen Sun

**Affiliations:** 1 The Centre for Applied Aquatic Genomics, Chinese Academy of Fishery Sciences, Beijing, 100141, China; 2 College of Fisheries and Life Science, Shanghai Ocean University, Shanghai, 201306, China; 3 Heilongjiang Fisheries Research Institute, Chinese Academy of Fishery Sciences, Harbin, 150070, China; 4 College of Life Science, Tianjin Normal University, Tianjin, 300387, China

## Abstract

**Background:**

Common carp (*Cyprinus carpio*), a member of Cyprinidae, is the third most important aquaculture species in the world with an annual global production of 3.4 million metric tons, accounting for nearly 14% of the all freshwater aquaculture production in the world. Apparently genomic resources are needed for this species in order to study its performance and production traits. In spite of much progress, no physical maps have been available for common carp. The objective of this project was to generate a BAC-based physical map using fluorescent restriction fingerprinting.

**Result:**

The first generation of common carp physical map was constructed using four- color High Information Content Fingerprinting (HICF). A total of 72,158 BAC clones were analyzed that generated 67,493 valid fingerprints (5.5 × genome coverage). These BAC clones were assembled into 3,696 contigs with the average length of 476 kb and a N50 length of 688 kb, representing approximately 1.76 Gb of the common carp genome. The largest contig contained 171 BAC clones with the physical length of 3.12 Mb. There are 761 contigs longer than the N50, and these contigs should be the most useful resource for future integrations with linkage map and whole genome sequence assembly. The common carp physical map is available at http://genomics.cafs.ac.cn/fpc/WebAGCoL/Carp/WebFPC/.

**Conclusion:**

The reported common carp physical map is the first physical map of the common carp genome. It should be a valuable genome resource facilitating whole genome sequence assembly and characterization of position-based genes important for aquaculture traits.

## Background

Common carp (*Cyprinus carpio*), a member of Cyprinidae, is the third most important aquaculture species in the world with an annual global production of 3.4 million metric tons, accounting for nearly 14% of the all freshwater aquaculture production in the world [1]. Common carp is mainly cultured in Eurasia continent with a culture history of several thousand years, and it was introduced into Africa and America some two centuries ago. In addition to its aquaculture importance, common carp is also considered as a model species for studies on ecology [2], environmental toxicology [3,4], development [5], immunology [6], evolutionary genomics [7], nutrition [8], and physiology [3]. As such, great interests exist to generate its genetic and genomic resources. Significant progress has been made recently including a large number of polymorphic genetic markers [6,9-11], linkage maps [12,13], a large number of ESTs (unpublished), a bacterial artificial chromosome (BAC) library [14], a large dataset of BAC-end sequences (BES) [15], and cDNA microarrays [16]. Some of these genomic resources have been used to analyze important genes [17] and quantitative trait loci (QTL) related to various economic traits such as growth rate, cold-tolerance, muscle quality, and amino acid content [18,19]. However, no physical maps have been constructed, hindering the progress of whole genome sequencing project as well as genetic improvement programs.

Common carp has a genome size of 1.6-2.0 Gb, as estimated from flow cytometry [20-22]. This is significantly larger than its closely related grass carp (1 Gb). Along with its large genome size, common carp has twice as many chromosomes as most other cyprinid fishes, making many to believe that an additional round of whole genome duplication (4R) may have occurred 50 Myr ago [23-25]. Such potential tetraploidization could add significant challenges to the whole genome sequencing project for common carp, a project currently in progress. Clearly, a physical map is demanded for scaffolding the small sequence contigs into scaffolds, and eventually into chromosome-scale sequence assemblies.

Physical maps have been proven as an important genome resource. A high quality physical map is very useful to understanding of genome structure and organization, and to positional cloning of genes associating to economically important traits. For genome sequencing projects, especially those using the high throughput next generation sequencing platforms, a high quality physical map and enough BAC end sequences are required to make the genome assembly accurately[26-29]. In addition, physical map could be integrated with linkage map by either mapping BAC-anchored genetic markers into linkage map or locating markers of linkage map on physical map contigs. The integrated map could be used in comparative mapping and genomic analysis of closely related species and enhance the understanding of unsequenced genomes [28,30].

In the past decade, several physical maps have been constructed in aquaculture ray-finned fishes including Nile tilapia (*Oreochromis niloticus*) [31], Atlantic salmon (*Salmo salar*) [32], channel catfish (*Ictalurus punctatus*) [33,34], rainbow trout (*Oncorhynchus mykiss*) [35] and Asian sea bass (*Lates calcarifer*) [36]. Here we report the first BAC-based physical map of the common carp genome.

## Results and Discussion

### BAC fingerprinting and contig assembly

**Figure 1:**
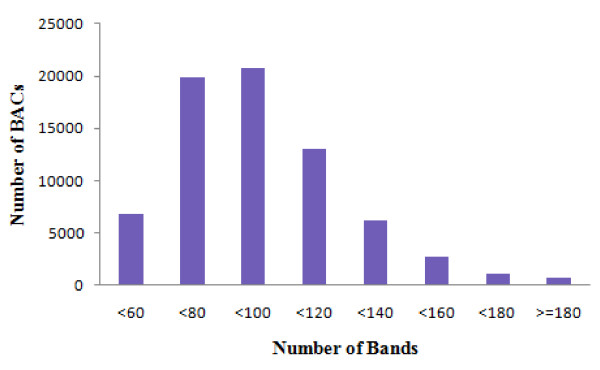
**Bands distribution in BAC fingerprints of the common carp**.

Four-color high information content fingerprinting (HICF) [37] was used to generate the restriction fingerprints of all BAC clones from the common carp BAC library. A total of 89,088 BAC clones, representing around 7.3-fold coverage of the common carp genome, were processed. Of these processed BAC clones, a total of 72,158 (81% success) fingerprints were used for the construction of the physical map after removal of low quality fingerprints using FPminer 2.1 [38]. These fingerprints represented approximately 5.9-fold coverage of the common carp genome (Table 1). On average, each BAC clone contained 98.7 restriction bands, with a range of 60 to 120 bands from most of the BACs (Figure 1). Each band represented approximate 1.4 kb on average, as assessed from the average insert size (141 kb) of the BAC library [14].

**Table 1:** Statistics of the physical map assembly of the common carp

Total number of BAC clones fingerprinted	89,088	~7.3× genome equivalent
Valid fingerprints for FPC assembly	72,158	~5.9× genome coverage
Total number of contigs assembled	3696	
Clones contained in the 3696 contigs	67,493	~5.5× genome coverage
Average BAC clones per contig	18.26	
Average contig size in consensus bands (CB)	334	
Estimated average contig size (kb)	476	
Estimated N50 contig size (kb)	688	
Number of Q-contigs	884	
Number of Q-clones	1448	
Number of singletons	4665	
Average insert size of the BAC library (kb)	141	
Average number of bands per fingerprinted BAC clone	98.7	
Average size each band represents (kb)	1.428	
Total number of bands included in the contigs	1,234,511	18.2 bands per BAC clone in the consensus map
Total physical length of assembled contigs	1.76 Gb	~1× genome size

**Figure 2:**
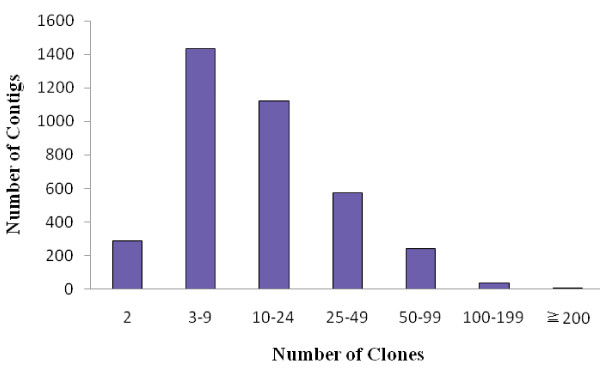
**Distribution of BAC clones in contigs**.

The 72,158 valid fingerprints were used for BAC contig assembly. A total of 67,493 BAC clones, representing 5.5-fold coverage of common carp genome, were assembled into 3,696 contigs. There were 4,665 unassembled BAC clones remaining as singletons. Each contig contains 18.3 BAC clones, with an average length of 476 kb (Table 1). The contig size distribution is shown in Figure 2. The largest contig contains 171 BAC clones with the physical length of 3.12 Mb. The N50 length of this assembly is 688 kb. There are 761 contigs longer than the N50, serving as the most useful resource for future integration with linkage map and whole genome sequence assembly.

There are a total of 1,234,511 consensus bands (CB) in this assembly, representing approximate 1.76 Gb of the common carp genome (1,234,511 CB × 1.428 kb per CB). Each BAC in the contigs contributes 18.2 distinct bands or 26 kb linear length to the assembly on average. The physical length of all the assembled BAC contigs is slightly longer than our commonly used estimation (1.7 Gb) of the genome size of common carp, but shorter than the estimation of Ojima and Yamamoto [20]. We believe that the summed length of all BAC contigs would be shorter than the real genome size as single BAC library cannot possibly cover 100% of the genome, because there would be some missing genomic regions caused by restriction enzyme bias, leaving gaps in the assembled physical map. However, a real BAC contig could be split into two contigs or more when we use assembly parameters of high stringency, especially for those genome regions with higher levels of heterozygosity.

Questionable clones (Q-clones) generally result from one or several false positive overlaps during physical map assembly. Sometimes, FPC may not be able to assign an appropriate linear order to a specific BAC clone on the consensus map, and marked it as a Q-clone. In this study, the function DQer were used to break up all contigs containing over 15% Q-clones after several rounds of end-to-end merging and single-to-end merging with lowered stringency of cutoff values progressively.

There were a total of 2,812 Q-clone-free contigs (76.1%) and 884 Q-contigs in the final version of the assembly. In the 884 Q-contigs, a total of 1,448 Q-clones were presented. However, vast majority of these Q-contigs only contained five Q-clones or less. There were only 12 Q-contigs (0.3%) containing more than 5 Q-clones (Table 2). The questionable clones could be generated by inconsistency in enzyme digestion, contamination, doubled peaks and possible chimeric contigs during the assembly. In spite of these technical effects, Q-clones could be also caused by the duplication status of common carp genome. Teleost fish genomes are well known for their whole genome duplication events, and high level of tandem and segmental gene duplications had been observed in teleost species. As common carp may have had an additional round of whole genome duplication (4R). High level of genome duplications would certainly add complexities for physical map assembly and possibly produce Q-clones.

**Table 2:** Distribution of Q-clones in assembled contigs

Number of contigs	Q-clones/contig	Percentage of all contigs
2812	0	76.1
552	1	14.9
188	2	5.1
80	3	2.2
34	4	0.9
18	5	0.5
12	> 5	0.3

Note that 76.1% of the contigs are free of Q-clones, and most Q-clones are involved in a small number of contigs.

The physical map of common carp is accessed through the web-based FPC viewer at http://genomics.cafs.ac.cn/fpc/WebAGCoL/Carp/WebFPC/.

### Assessment of the physical map

**Figure 3:**
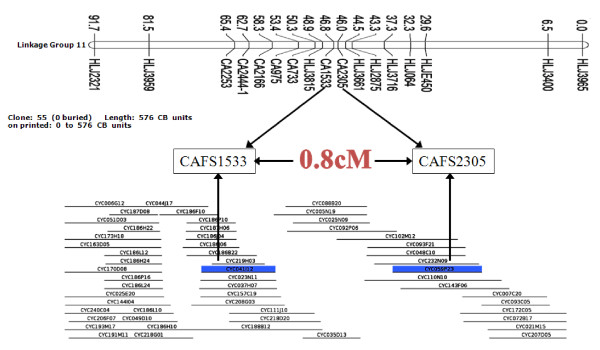
**Example of common carp contig anchored to a genetic linkage group using microsatellites isolated from BAC end sequences**. The contig 251, containing 55 BAC clones, has two microsatellite markers (CAFS1533 and CAFS2305) mapped to linkage group 11. The linkage distance between CAFS1533 and CAFS2305 is 0.8 cM.

Three approaches were used to assess the reliability of the physical map assembly for common carp. First, eight contigs were randomly selected which contained end-to-end merging junction. The primers were designed from BAC end sequence on one side of the junction site, and used in the PCR on both BAC clones of the junction. The results showed that PCR can successfully amplify positive products on the junctions of eight selected contigs, indicating that both BAC clones on the junction site were truly overlapped in the common carp genome (Table 3). Since the junction sites were generated by end to end merging with looser stringency, the reliability of the whole contig was then proved. A second approach was also used to assess the physical map assembly from randomly selected contigs. Briefly, eighteen contigs with various lengths were selected randomly. PCR primers were designed from BAC end sequences, and PCR reactions were performed on all BAC clones. For those long contigs, multiple pairs of primers were designed and used, if necessary, to cover all BAC clones in the contig. If all the BAC clones truly belong to the contig, they should be identified by PCR reactions in the contig, thereby confirming the contig. As shown in Table 4 all of the BAC clones in surveyed eighteen contigs could be positively identified by PCR assays, providing strong evidence for the high reliability of the physical map of common carp. The third approach was used to evaluate the physical map assembly by mapping physical map contigs to common carp linkage map (Zhang, et al, unpublished). The unpublished linkage map contained 858 microsatellite markers, including 271 BAC-derived microsatellite markers, which make it good resource for the physical map assessment. A total of 179 BAC-derived microsatellite markers had been located on both physical map contigs and linkage map which could serve as anchors for map integration. A total of 7 contigs had been identified that mapped on 7 distinct linkage groups. The genetic distances of two anchor points on the linkage map were ranged from 0.80 cM to 6.39 cM (Table 5). Thus, all of the 7 physical map contigs had been validated using linkage analysis, further validating the physical map assembly of common carp. Figure 3 was an example that common carp physical map contig 251 was anchored to a genetic linkage group 11 by using BAC-anchored microsatellite markers. More contigs could be mapped on linkage map by developing and anchoring more contig-derived markers on common carp linkage map, which will lead to comprehensive integration of physical and linkage maps gradually.

**Table 3:** Assessment of overlapping reliability at end to end merging points by using PCR

Clones overlapping each other		cutoff value of end merging	Positive(+)/Negative(-)
CYC054L01	CYC121J09	1e-40	+
CYC204K23	CYC054L01	1e-40	+
CYC027I24	CYC203H04	8e-37	+
CYC096 P15	CYC148K22	4e-36	+
CYC016K05	CYC207J08	5e-25	+
CYC013P01	CYC240P24	1e-21	+
CYC086L08	CYC118D14	4e-36	+
CYC055K17	CYC098L15	2e-28	+

Note that positive (+) indicates two overlapping clones are truly overlapped at the end-to-end merging point.

**Table 4:** Assessment of assembly reliability on randomly selected contigs by using PCR with primers designed from BAC end sequences

Contig ID	Number of primer pairs	Number of Clones	Number of Positive Clones	Contig assembly completely validated
2042	15	65	65	Yes
113	12	85	85	Yes
2092	8	34	34	Yes
348	2	11	11	Yes
4368	4	11	11	Yes
1494	4	25	25	Yes
2220	4	14	14	Yes
4074	2	10	10	Yes
1258	2	13	13	Yes
6064	2	13	13	Yes
3806	4	17	17	Yes
4498	4	13	13	Yes
677	6	37	37	Yes
2995	4	24	24	Yes
3682	4	22	22	Yes
5929	2	7	7	Yes
3097	2	12	12	Yes
984	2	12	12	Yes

**Table 5:** Validation of physical map assembly by linkage mapping of microsatellites isolated from clones in the common carp physical map.

Contig ID	Number of BAC Clones	Contig Length (kb)	Genetic Distance (cM)	Number of Markers	Linkage Group ID
251	55	822	0.80	2	11
285	30	765	1.55	2	2
633	11	237	3.35	2	16
1769	25	510	4.07	2	4
3108	19	590	1.73	2	1
3749	28	520	6.39	2	36
4017	20	550	1.33	2	3

## Conclusion

Here we reported the construction of the first physical map of the common carp genome. The physical map was constructed with valid fingerprints of 67,493 clones (5.5 × genome coverage). The physical map can be accessed at http://genomics.cafs.ac.cn/fpc/WebAGCoL/Carp/WebFPC/. This physical map contained 3,696 contigs with a N50 length of 688 kb. The consensus length of assembled contigs was 1.76 Gb, consistent with the estimated genome size of common carp (1.7 Gb-2.0 Gb). The assembly was validated by using PCR assays on randomly selected contigs and mapping physical map contigs on linkage map. This physical map should be useful for various genome projects of common carp, especially for the currently ongoing whole genome sequencing project of carp.

## Methods

### BAC library

The *Hin*d III BAC library of common carp used for the construction of the physical map was previously reported [14]. Briefly, the library was made from a female common carp with a total of 92,160 recombinant clones and an average insert size of 141 kb. This library represented approximately 7.6-fold genome coverage of the common carp genome.

### BAC DNA isolation and fingerprinting

BAC clones were inoculated into four 96 deep-well culturing plates using a 96-pin replicator (V&P Scientific, San Diego, CA, USA). Each well of the 96 deep-well culturing plates contained 1.2 ml 2×YT medium and 12.5 μg/ml chloramphenicol. The deep-well culturing plates were then covered with air permeable seals (Excel Scientific, Victorville, CA, USA) and incubated at 37°C with 300 rpm shaking for 20 hours. BAC DNA was then isolated using a modified alkaline method with lysate clarification using Fritted Filter Plate (NUNC, Roskilde, Denmark). BAC DNA was resuspended in 50 μl of milliQ water in 96-well plates and stored at -20°C before use.

Twenty μl BAC DNA of each BAC clone was digested by *Bam*HI, *Eco*RI, *Xba*I, *Xho*I, and *Hae*III restriction endonucleases (New England Biolabs, Ipswich, MA, USA) at 37°C for three hours simultaneously, and then end-labeled using SNaPshot Multiplex kit (Life technologies, Foster City, CA, USA), according to manufacturer's instructions. The 6-bp cutter restriction endonucleases *Eco*RI (G'AATTC), *Xba*I (T'CTAGA), *Bam*HI (G'GATCC), *Xho*I (C'TCGAG) generate 5'-protruding ends allowing differentially fluorescence labeled A, C, G, and T to be incorporated at the 3' ends of fingerprints while the 4-bp cutter *Hae*III cleave the fragments to small segments making them suitable for analysis using an automated sequencer [37]. The labeled BAC fragments were precipitated by using pre-chilled 100% ethanol following by washing with 70% ethanol, then suspended in 10 μl Hi-Di Formamide and analyzed with GeneScan 500 LIZ Size Standard on 3730XL DNA Analyzer (Life technologies).

### Fingerprint collection and processing

The fragment sizes in each BAC fingerprint were collected by the Data Collection program on the ABI 3730XL Genetic Analyzer, then processed by software FPminer 2.1 [38]. Briefly, the fragment size calling was conducted using automatic algorithm in FPminer. Several quality control steps were applied to the fingerprints: the empty wells were removed; the off-scale fragments with peak height greater than 6,000 relative fluorescent units (RFU) were removed; the fingerprints with fewer than 50 or more than 250 fragments were removed. Cross-contamination check was also conducted on FPminer to remove potential contaminated clones. In addition, the fingerprints having greater than 60 fragments of any single fluorescent color were also considered as contaminated clones and removed. Vector fragments and high frequency fragments were identified by fragment frequency analysis and then removed in FPminer. The *sizes *files were then output from FPminer for contig assembly in FPC program (http://www.agcol.arizona.edu/software/fpc).

### Contig assembly

The program FPC version 9.3 was used to assemble the BAC fingerprinting data into BAC contigs. FPC parameters were adjusted for the HICF method as described in the tutorials. The size tolerance was set at 0.4 bp, and Sulston score cutoff was initially set as 1e-40. After the first round of assembly, the DQer function was performed to break down all contigs more than 15% of Q clones to eliminate false assembly. Several rounds of end-to-end merging with consecutive reductions of the Sulston score cutoff stringency at 1e-15 were then performed, and followed by single-to-end merging until the final cutoff of 1e-15 was reached.

### Contig quality assessment using PCR method

BAC contigs were randomly selected, and BAC-end sequences on those selected contigs were used to develop primers for contig validation and reliability examination. Briefly, all BAC clones on the selected contigs were picked from stocking plates and inoculated into culturing plates. BAC DNA was then extracted using alkaline method as we described above. PCR reactions with primers from specific contig were conducted on all BAC clones of the contig in 25 μl solution containing 10 ng BAC DNA, 1×PCR buffer, 100 μmol of each dNTPs, 0.2 μmol forward primer, 0.2 μmol reverse primer and 1 U of Taq DNA polymerase (Fermentas, Glen Burnie, Maryland, USA) on ABI 9700 thermal cycler (Life Technologies) under the following cycling conditions: initial denaturation at 95°C for 3 min; then 35 cycles of 94°C for 30 sec, 55°C for 30 sec and 72°C for 45 sec; final extension at 72°C for 5 min. All primers were listed in Additional file 1 Table S1. PCR products were then analyzed to detect positive BAC clones using electrophoresis on 1.2% agarose gel. The BAC clones that supported positive PCR amplification with a single pair of PCR primers were considered to be overlapped.

### Contig validation using BAC-anchored microsatellite markers on linkage map

Microsatellite markers were previously developed from BAC end sequences[15] and genotyped in a F1 common carp family for linkage mapping (Zhang *et al*, unpublished). The linkage map contained 271 BAC-derived microsatellite markers, which could serve anchor points for physical and linkage map integration. Physical map contigs containing at least one anchor microsatellite markers were then mapped to linkage map. The contigs harboring two or more BAC-anchored microsatellite markers were collected for assembly assessment. Microsatellite markers on one physical map contig should be also mapped to one linkage group with reasonable genetic distance if physical map was assembled correctly.

## Authors' contributions

PX designed and supervised the physical mapping project, and drafted the manuscript. WJ worked on data collection and physical map assembly. JTW, YL and RC participated in BAC culture and DNA extraction. ZZ worked on BAC library manipulation. YZ participated in microsatellite identification and linkage analysis. PJ and JL worked on bioinformatics analysis and WebFPC. XS supervised the common carp genome project. All authors read and approved the final manuscript.

## Supplementary Material

Additional file 1 **Table S1**. All primers used for the assessment of common carp physical map. Click here for file
