# Supplementary material for: Generation of the first BAC-based physical map of the common carp genome
Source: BMC Genomics. 2011 Nov 2;12:537. doi: 10.1186/1471-2164-12-537 (PMC3221725; doi:10.1186/1471-2164-12-537)
Supplement: Additional file 1 — Table S1. All primers used for the assessment of common carp physical map. [file 1471-2164-12-537-S1.DOC]

Table S1. All primers used for the assessment of common carp physical map.

| Primer | Contig | Forward primer | Reverse primer |
| --- | --- | --- | --- |
| 032N08.f | Contig2042 | GCCACTTCACTTTGCCATC | TTGGTTGGCTGCTTGTCAT |
| 032N08.r | Contig2042 | CTCTGTGCCTGACTGATGC | GGGTTGTGTTAGTGGCAGG |
| 023K15.f | Contig2042 | CCACTAAACAACGCTAACG | TGTGATGCTTTTTCCTTGC |
| 023K15.r | Contig2042 | TAACCCCTTTTTCCATCG | ACAGAATGGTAGTGTAAAT |
| 092F12.f | Contig2042 | ACACCACAATCCCACTCCA | TTCACGCAAACATAACCGA |
| 061N10.f | Contig2042 | CACAGTGAAGGAGAAGCGT | CTCTCTTTTATTCACGGGC |
| 061N10.r | Contig2042 | CAGAGAGTGACGGGCTAAC | AAGTGTAAAAAGCCAAGCG |
| 021G04.f | Contig2042 | TGTAGTCGCTCTTTTCCAT | GAACGAAGAAGAGCAAAGG |
| 021G04.r | Contig2042 | GAATCAAAGATGTTGGCAG | CATCCACACTAACTCCCTG |
| 040O10.r | Contig2042 | GTCAGTGGACGGAAGCC | CGCACTCATTGTTCACG |
| 040O10.f | Contig2042 | TTCCGAACACACTCCGC | ATTCCCTTCTGCCCTCC |
| 015N17.r | Contig2042 | TAAGGCAGTGACACAAGCA | GCTGATGGAATGTGTCTGT |
| 015N17.f | Contig2042 | AACACAACTCCCTCCTGCT | CATCAAAATGAAAGTGCCA |
| 076M05.r | Contig2042 | AGTAGGCAGGTGGCAGAT | GGCAAATCCCACAAACTGA |
| 076M05.f | Contig2042 | TGTGTTCCCTAAAATCCAT | AGAATGACGAGTGGCAAGA |
| 028J10.f | Contig113 | TCATACTGTTGATTCCACC | ATGTTTTTGTATGTGTGCC |
| 028J10.r | Contig113 | TTGACACCCTGAGGAGCAT | TGGCACAAACTTGAGAACT |
| 091K18.f | Contig113 | GACTTGAATCATCCCTGTG | TTCAGGGACCAATAGAGGA |
| 091K18.r | Contig113 | GACTGGCTAATAATGGTGC | GAACAAGCAGCCTAATCCT |
| 031K19.f | Contig113 | CTGTCAGTGTTGGGAAGTA | AACTCCTGAAACATTGGTG |
| 031K19.r | Contig113 | CCAATAAAAAGTGGCAGGC | GGCACCCTACTGAAATCTC |
| 019N12.f | Contig113 | GATGTTCTTTCATACCTTG | GTTCACCTAAATACAAAGT |
| 019N12.r | Contig113 | ATGAGCAGGAGATGGATTA | GGCAGGAACACATTTGAAC |
| 065F02.r | Contig113 | TGGTTTGTGTTCTTGCTCA | GAGCGTCTGAACGAAAGGT |
| 065F02.f | Contig113 | AGAGCAGTGGCTGAGAGAT | AGATTAGCATCGTCCAAGT |
| 056A08.r | Contig113 | ATTGTGTGTGAGGATGTTG | GACAAACAATACCTTCCTT |
| 056A08.f | Contig113 | TTGAAAAAAGGCTGAATGA | GATTGGGGGTTTATGTGAA |
| 030P09.f | Contig2092 | ATGTTGCGTTGACTTCTTG | TTACCAGCAGTCTGAAGCA |
| 030P09.r | Contig2092 | GCTGAGAGAGGAGATTTGT | CTGGTTAGAGTGGAGGCAT |
| 093E16.f | Contig2092 | ATTCATTGGAACAAGGGTG | TAAAGTTGGCGAAGAGCAG |
| 093E16.r | Contig2092 | GTGATGATGGTCAACAGTC | TTGTCAGGAATAAAAATAC |
| 056B19.f | Contig2092 | AAGGAAATACTCCCCCCAG | GTCGGAAAGTGTTGTGGTT |
| 056B19.r | Contig2092 | CTGTGTTAGTTCTGCTGGC | ATCTCAGTGCTATCTTTGC |
| 038K04.r | Contig2092 | TTAGAGCCTTTGAACAGTC | CTCTTACAGGGCTTCTCTT |
| 038K04.f | Contig2092 | GAGCCCAAGGGACAGTTAT | ATTTGGGGGTTAGGCAGAT |
| 045J02.f | Contig348 | AAGGATTTCTGAAGGATTG | CTGTGTCACTGAGAGGGAT |
| 045J02.r | Contig348 | TGAAGAGAAACTGTGGTGG | TGAAGACAAAACCACAAGG |
| 023D18.f | Contig4368 | TGAGGCTGAGTAAAGGAAG | TACTGAAGTGTCTCCCCGC |
| 023D18.r | Contig4368 | AGCAAAACAAGTCTAACGC | TTCCCATAAATAAACAGCA |
| 069H01.r | Contig4368 | CCATCAACGCCAATAAAGA | CATAGATGCTGGGCTTGGT |
| 069H01.f | Contig4368 | CAATGAGAGGATAACCCAG | CAGAGTCTTTTGTGATTGG |
| 099E19.f | Contig1494 | AAAACCACACAAGGCACAG | GGGTGCTAACATTTTCAAC |
| 099E19.r | Contig1494 | GACCCCACAGAGAGACAAT | TCATTGGACCTACAGTTGC |
| 025N20.r | Contig1494 | TTTCTCCCTCATTTGTAAC | GAGAAAGAGAGGGAGTAAT |
| 025N20.f | Contig1494 | TATTATGGTTATTTTGCGT | TACTTTGTTGAGAACCCAT |
| 088G17.f | Contig2220 | TAAAATCCTCTCCGTCCAC | GGTGAGTGAGTGCTTCCTG |
| 088G17.r | Contig2220 | CGGTCTCAGAAGTGGCATC | TTCGTATTTGAAAACAGCC |
| 087P19.r | Contig2220 | CTGAAGAAATCCCAACTGT | TGGGTCAGGACAAAGCACT |
| 087P19.f | Contig2220 | AAGGATTATTAGGAACACC | GACATTCAGTTTGGAGTTC |
| 081C14.f | Contig4074 | TAACCTCTTTGTGCTTTCT | CAGACAGTGAAACAATGGA |
| 081C14.r | Contig4074 | GGATTGGAAAATACCGTGT | ACTCCCCCCTCTCCTTCTG |
| 036E03.f | Contig1258 | CGAGAGTTAAAGGGCGTAT | GCAGGTGAGTTCAGGAGAG |
| 036E03.r | Contig1258 | TGAGAAATGTTGACTTGGC | AGGGAGCCAGAGAGTCACC |
| 020H10.f | Contig6064 | TTATCACCACATTTCACCT | TTTTAGTTGCTGAAGGATT |
| 020H10.r | Contig6064 | CAAATCCCCCAAGACAAAT | GGTTAGATGTGGACTGGTG |
| 018M22.f | Contig3806 | TTTGGCATCATCATTACCT | TGACCCTAAACACAGCAAT |
| 018M22.r | Contig3806 | CACTTGTGACAGCGGGT | TTTTTGTGAGGAACAGACT |
| 096F18.r | Contig3806 | AATGTATGATGGCAGTAGC | CTTTACATTTCTTTGCCCT |
| 096F18.f | Contig3806 | ATCTGTCTACTTGGGTGGT | CAGCATCTGTGCCTCTTAG |
| 036C22.f | Contig4498 | AGATTTTGCGAAACTCACG | TCTGTTAGCCACTGGTTTG |
| 036C22.r | Contig4498 | GCTGACCTGACTTGAACT | TAGAGACAGGTGACGGAT |
| 081O12.r | Contig4498 | CCAAAACATCTTGTCAGGC | TTTCCAAACTTCTGACCTG |
| 081O12.f | Contig4498 | TCACCCCCTCACAGTTATC | AAAGGATGGCTCCAAAATG |
| 073B17.f | Contig677 | TTTTTCCTGGCTATTGACA | CTAACTGCGTTCATCTCAT |
| 073B17.r | Contig677 | TTCCCTAAATGTTTCCCAC | CGGTTATCTGTTTTTGTCC |
| 024P16.r | Contig677 | GCCTTTGAGTAGAAGTGAA | TGTGTATGTCTTACTGCGG |
| 024P16.f | Contig677 | TATGTGTGCGTGAGAATGG | CTGAGGACTGTATGATGCC |
| 084J07.r | Contig677 | CATCTTTCATGTGAGCAAG | AGCTTCAAAGCATGACATG |
| 084J07.f | Contig677 | TTCACTGTTATCGGTCCAC | TTGTAAGGCAGAATGATGT |
| 087O05.f | Contig2995 | GAAAATGTGCTGGCTTGTG | TTGTCACTCATCCTTCCAG |
| 087O05.r | Contig2995 | CACTTCAATAATGCCTCTG | CAACTTATGGATACCCTTA |
| 056D15.r | Contig2995 | TTAGCCAGTCTGTCTTCTT | GGATGTCTAAGTGGAGTCT |
| 056D15.f | Contig2995 | ATGGGGGTAAGTGAATAAT | TCACACTAAAAACCAGGGC |
| 057A01.f | Contig3682 | GCTGTTGCCAATAGGTGTG | GCTGTTGCCAATAGGTGTG |
| 057A01.r | Contig3682 | TAGCAGACTGACAGCAAG | AGTCACCTTTTTTCGGC |
| 022B05.r | Contig3682 | TCAATCAAACAAATGGCGT | GAACAATCTCAACCAATGC |
| 022B05.f | Contig3682 | TGGAGGTGTTTGGTTATGC | CTCCTCCAGTGACTCCGAT |
| 025L18.f | Contig5929 | ACATTTTTTGCTTTTGGAT | TCCTTATGAGCAAAGAACT |
| 025L18.r | Contig5929 | AGCAACCTTCTGTGAGCC | CTCACCTTCCCCTCTTCTC |
| 045K08.f | Contig3097 | AACCCACAAGCAGAAAAGAC | CCAACACTCACACCAACAG |
| 045K08.r | Contig3097 | TTTGAAACACAACTGAAGAC | AGTGACTGTGCTGAAAAAC |
| 039A09.f | Contig984 | CTTTAGAGATGTTCTTGGC | GTGAACCTAAAAACAGCCT |
| 039A09.r | Contig984 | GAGTGCGAGTTGTGTCAG | GCCACTGTGACAAGAAATC |
| 055K17.r | Contig110 | CAAATCCATTAGCCCTTC | TCTTCTTTGGACATTTTGG |
| 055K17.f | Contig110 | AAGCCAATAGATGGTGTTC | ACAGTTTACTCAGGCACCA |
| 013P01.r | Contig175 | AAACAGCCATTGGTCATAC | CTATTTGAGCAGATGAGGC |
| 013P01.f | Contig175 | TTTGGCAGCGTTGTAAGAC | TAAAGGCTTCTTGTTGGAG |
| 016K05.r | Contig642 | TAACGCTCACTGACCGAC | GTGAAGTCCATCGATCTG |
| 016K05.f | Contig642 | ATTTCGGTTTGGATTCACT | TTAGCAAAGTGCCACAAAG |
| 027I24.r | Contig200 | CATTGGTTTGATGAGGTGT | GCGAACTTTACAACCTACC |
| 027I24.f | Contig200 | CGTTCAGATAGTGTGCTTG | CTGAATGACAGGAAAGGAT |
| 054L01.r | Contig200 | TCCTGATGTTTATGTGAGC | GAAGGTTTCAGATGTTTGG |
| 063I21.r | Contig942 | TGTTTTACAGCGAATACCG | GATTTCTGCTCTCTTTCCG |
| 063I21.f | Contig942 | ATGATAGTCTGGTGGCGTG | GAAGCAGCAGACAACAATA |
| 080D01.r | Contig942 | ATGTCTGGGTGACTTGTTG | GCATCTTACCTTCGCTGTG |
| 080D01.f | Contig942 | GGCTTTTGTTGATGTTGTTC | CATTACTCCAGTCTTCACG |
| 086L08.r | Contig555 | GTTAGAGGAGTAGCAGGCA | ACAAAGGTCTTATGGGTTC |
| 086L08.f | Contig555 | AAGTGTGTTGGGATGGATA | TGAAAAATCTAACACGACT |
| 096P15.r | Contig1281 | GCGAAATACTAACAGCATC | GAACCTGATGTCAAGCAAC |
| 096P15.f | Contig1281 | AAGTTCACAAAATGGGAGC | TGAACTTCTATCCCTCTGG |
